# Harnessing the nutraceutical and therapeutic potential of *Allium* spp.: current insights and future directions

**DOI:** 10.3389/fnut.2024.1497953

**Published:** 2024-11-14

**Authors:** Kalyani Gorrepati, Ram Krishna, Saurabh Singh, Dhananjay V. Shirsat, P.S. Soumia, Vijay Mahajan

**Affiliations:** ^1^ICAR-Directorate of Onion and Garlic Research, Rajgurunagar, Pune, India; ^2^ICAR-Indian Institute of Vegetable Research, Varanasi, India; ^3^Institute of Environment and Sustainable Development, Banaras Hindu University, Varanasi, India

**Keywords:** *Allium*, bioactive compounds, health benefits, nutraceuticals, therapeutic properties

## Abstract

Apart from the culinary usage, Alliums are known for their therapeutic potential since antiquity. Alliums contain diverse bioactive compounds such as, sulfur-containing compounds (allicin, diallyl sulfides), flavonoids, and saponins. These compounds have demonstrated a wide range of pharmacological actions, including antioxidant, anticancer, anti-inflammatory, antimicrobial, neuroprotective, cardioprotective activities and treatment of metabolic disorders such as diabetes and hyperlipidemia. Despite encouraging preclinical results, translating these findings into clinical practice remains difficult, necessitating more rigorous human trials and molecular research. One of the major constrain in enhancing the therapeutic efficacy of these bioactive compound is to develop large-scale extraction techniques besides improving their stability, solubility, and bioavailability. The current scenario urges to focus research on optimizing the bioavailability of these compounds, evaluate their synergistic effects with existing therapies, as well as their long-term safety. This perspective article provides a comprehensive overview of the therapeutic potential of *Allium* spp. and suggests the key avenues for future research aiming at realising their full clinical potential.

## Introduction

Alliums are well-known for their diverse medicinal and therapeutic properties and have been a part of various medicinal systems for centuries. The genus Allium belongs to the family Amaryllidaceae of the order Asparagales. More than 920 species have been reported in the Allium genus, of which garlic (*Allium sativum*), onion (*A. cepa and A. cepa* var. *aggregatum*), leek (*A. ampeloprasum*), chive (*A. schoenoprasum*), Welsh onion (*A. fistulosum*), and shallot (*A. ascalonicum*), being some well-known and widely cultivated species (1). Various parts of allium plant (i.e., leaves, flowers, pseudostem, bulb, and seeds) are consumed in different forms due to their versatility. The characteristic taste and smell of these species is attributed to certain bioactive compounds, including organosulfurs (allicin, diallyl disulfide), flavonoids (quercetin, kaempferol), saponins and other phytochemicals (2–5). Due to these versatile bioactive compounds, the consumption of Allium species has been associated to numerous health benefits, such as cardiovascular benefits, gastrointestinal health, antimicrobial and antiviral properties, anticancer potential, anti-inflammatory and antioxidant effect etc.

Globally, onion and garlic are the highly valued allium crops for the unique aroma and pungency. Beyond enhancing the flavour and taste, they act as functional foods with significant health benefits. Nevertheless, the demand for these crops are often unmet due to several factors. This urges for the need of an alternative source that provide nutritional diversity without compromising the taste and flavour. In addition, leeks and Welsh onions are utilized extensively in some parts of western and Asian countries. Therefore, as an alternative to the onion and garlic, underutilized alliums such as *Allium tuberosum, Allium fragrance, and Allium chinense* can be incorporated into the daily diet. Furthermore, these plants are ideal for kitchen gardens or small pots due to the ease in cultivation, require minimal space and low maintenance, making them perfect for nuclear families. These crops can be regenerated from their stubbles, as they only need to be planted once for ongoing harvests. Furthermore, Alliums are low in calories and rich in essential nutrients such as vitamins (such as vitamin C and B vitamins), minerals (such as potassium and selenium), and dietary fibers which play a pivotal role in enhancing the nutritional quality of diets besides their therapeutic properties. Due to their importance as a staple in both diet and medicine, reinforcing the proverb, “Let food be thy medicine and medicine be thy food.” With the advent of various technological advancements, the important bioactive compounds from these allium species have also been extracted, encapsulated and consumed as supplements for preventing and treating various lifestyle diseases. Despite the numerous health benefits of Allium species, there are some concerns regarding the stability, dosage, bioavailability and synergistic effect with other compounds. In this, we have tried to comprehend the information related to bioactive compounds and their diverse medicinal and therapeutic benefits.

## Bioactive compounds in *Allium* species

*Allium* spp. contain several bioactive compounds that exhibit a range of health-promoting properties, including antioxidant, anti-inflammatory, antimicrobial, and anticancer activities (Table 1). Among the various *Allium* spp., garlic is rich in vitamins, enzymes, organosulfur compounds (OSCs), phenolic compounds etc. OSCs like allicin in garlic, and thiosulfinates (isoalliin) in onions, are associated with anticancer, cardiovascular, and antimicrobial effects (6, 7). Likewise, phenolic compounds such as flavonoids are abundant in *Allium* spp. Quercetin in onions is associated with antioxidant, anti-obesity, anti-cancer, and cardiovascular benefits (8, 9). Alliums are also rich in non-structural carbohydrates like Fructans which acts as a prebiotics. For instance, onion contain 2.8% of fructooligosaccharides (FOS) whereas garlic contains 1.0% FOS (10). FOS aid in improving the gut health and enhancing mineral absorption, thereby supporting bone health and reducing atherosclerosis (11). Furthermore, garlic and onion are rich in minerals such as selenium (Se) that acts as a cofactor for various antioxidant enzymes (12). Although *A. tuberosum* has higher antioxidant activity in compared to *A. sativum* (13), it also protects against CCl4 induced liver injury (14). Meanwhile, consuming leek (*A. ampeloprasum*) can help reduce the risks of hypercholesterolemia, high blood pressure, arteriosclerosis, and platelet aggregation there by preventing cardiovascular diseases (15). Similarly, Alliums are rich in vitamins such as Vitamin C, K, B12 etc., which enhances the immune system (16).

**Table 1:** Medicinal properties of bioactive compounds in *Allium* spp. along with their mode of action.

Bioactive compound	Plant organ	Medicinal properties	Mode of action	Reference
Onion (*Allium cepa*)				
Quercetin	Bulb, bulb peel, foliage	Antioxidant, anti-inflammatory, anticancer	Inhibits free radical formation, reduces inflammation markers, induces apoptosis in cancer cells	(104–106)
Sulfur Compounds		Antimicrobial, cardiovascular health, anticancer	Inhibits microbial growth, reduces cholesterol, induces cancer cell death	(102, 107)
Saponins		Antimicrobial, cholesterol-lowering	Disrupts microbial cell membranes, inhibits cholesterol absorption in the intestines	(108, 109)
Flavonoids		Antioxidant, cardioprotective, anticancer	Scavenges free radicals, enhances nitric oxide production, inhibits cancer proliferation	(110–112)
Alk(en)yl Cysteine Sulfoxides		Antioxidant, antihypertensive, antiplatelet	Reduces oxidative stress, lowers blood pressure, inhibits platelet aggregation	(113, 114)
Garlic (*Allium sativum*)				
Allicin	Bulb, bulb peel, foliage	Antimicrobial, antioxidant, anti-inflammatory, antitumor	Inhibits bacterial enzyme activity, reduces oxidative stress, modulates inflammatory pathways	(115–118)
Ajoene		Antimicrobial, anticoagulant	Inhibits platelet aggregation, has antifungal properties	(116, 119, 120)
Sulfur Compounds (Diallyl Disulfide, Diallyl Sulfide, S-Allylcysteine)		Anti-cancer, Antioxidant, anti-inflammatory, cardioprotective, liver protective, neuroprotective	Lowers cholesterol and blood pressure level, has anti-inflammatory and anticancer properties. Induces apoptosis in cancer cells and inhibits tumor growth; neutralizes free radicals.	(121–123)
Chives (*Allium schoenoprasum*)				
Allicin	Pseudo stem, foliage	Antimicrobial, anti-inflammatory, antioxidant, cardiovascular benefits	Inhibits bacterial growth, reduces inflammation and oxidative stress.	(124–126)
Quercetin		Antioxidant, anti-inflammatory, antihistamine, anticancer, anti-diabetic	Scavenges free radicals, reduces inflammation, and inhibits histamine release. Inhibit cancer cell proliferation.	(14, 125)
Leek (*Allium ampeloprasum*)				
Allicin	Pseudo stem, foliage	Antimicrobial, anti-inflammatory, antioxidant	Inhibits microbial growth by disrupting cellular processes and has anti-inflammatory effects by reducing cytokine production.	(15, 127)
Quercetin		Anti-inflammatory, antioxidant, antihypertensive	Scavenges free radicals and inhibits inflammatory pathways, relaxes blood vessels, reduce blood pressure.	(128, 129)
Sulfides		Cardioprotective, anti-inflammatory	Enhance cardiovascular health by improving lipid profiles and reduce inflammation.	(129, 130)
Flavonoids		Antioxidant, anti-inflammatory, anticancer	Modulate cell signaling pathways, reduce oxidative stress, and inhibit cancer cell proliferation.	(131–133)
Welsh onion (*Allium fistulosum*)				
Quercetin	Pseudo stem, foliage	Antioxidant, Anti-inflammatory, Anticancer	Scavenges free radicals, inhibits inflammatory pathways, induces apoptosis in cancer cells	(39, 134–136)
Allicin		Antibacterial, Antifungal, Antioxidant	Inhibits microbial enzyme activities, reduces oxidative stress	(127, 137)
Sulfur Compounds		Cardioprotective, Anticancer, Detoxifying	Modulates detoxification enzymes, inhibits tumor growth, protects cardiovascular health	(138, 139)
Flavonoids		Anti-inflammatory, Antioxidant	Reduces inflammation, scavenges reactive oxygen species	(136)
Edible *Allium* spp.				
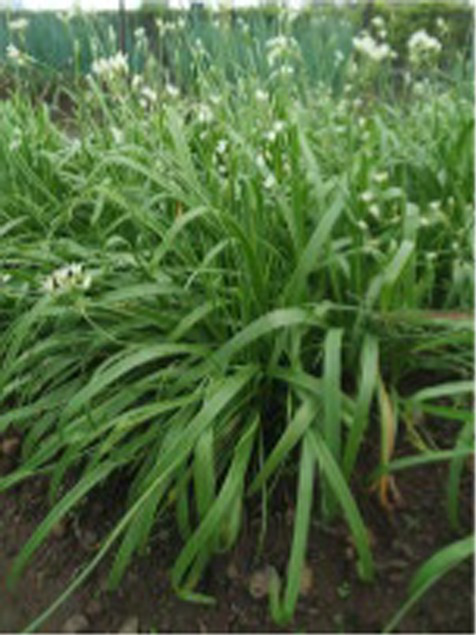 *Allium fragrance*	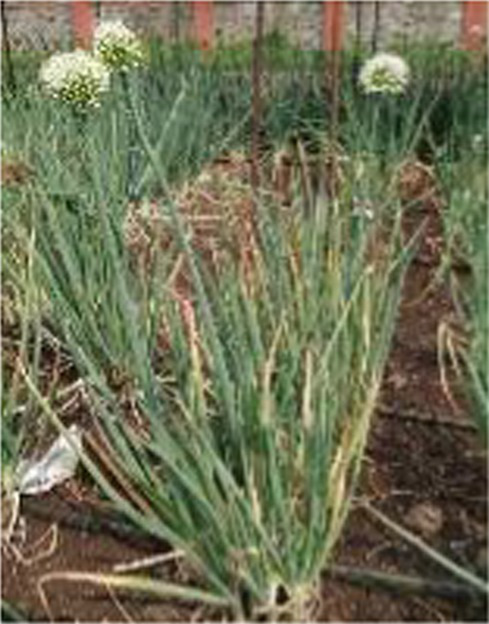 *Allium fistulosum*	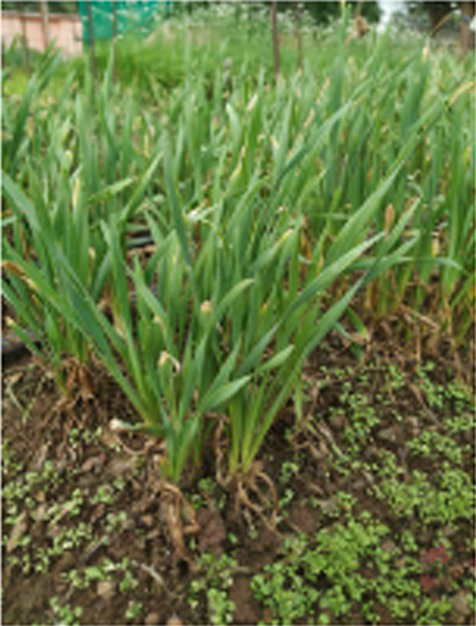 *Allium angulosum*	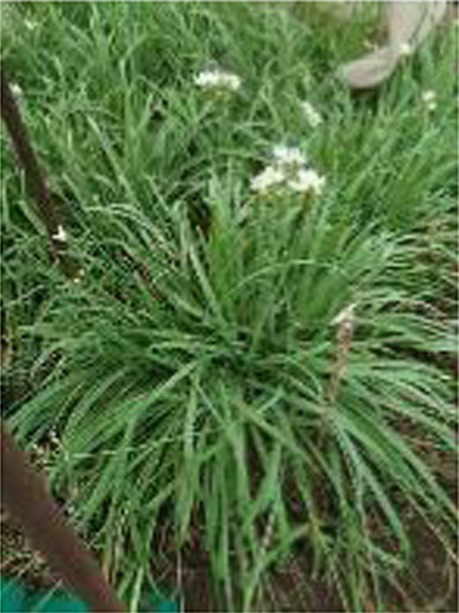 *Allium tuberosum*	
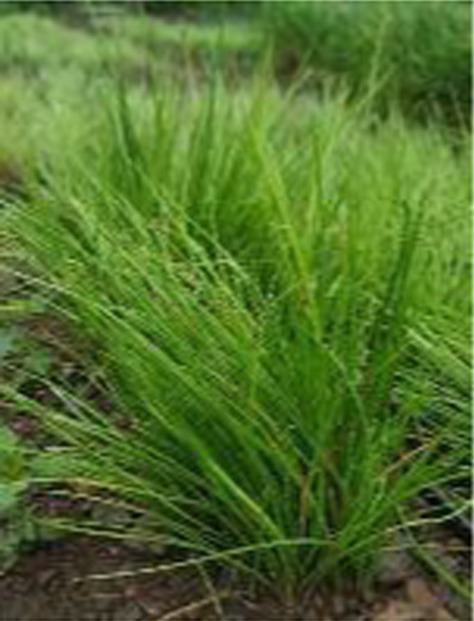 *Allium chinense*	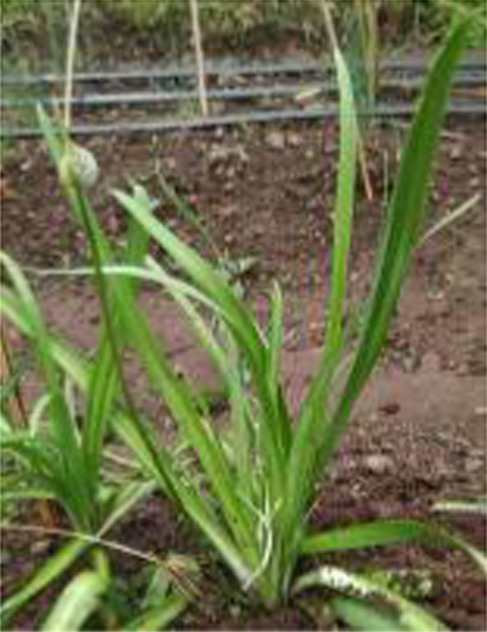 *Allium hookeri*	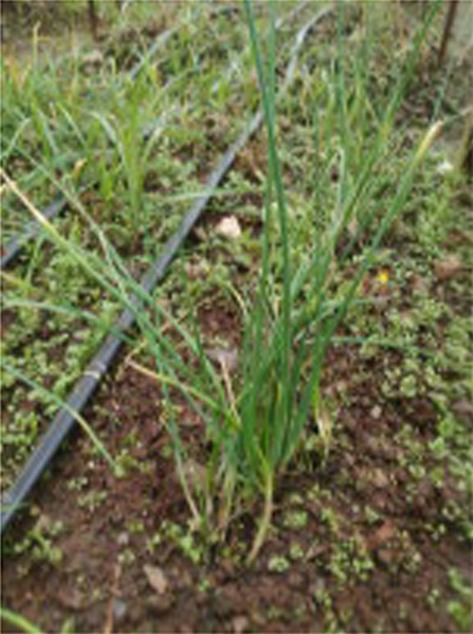 *Allium schoenoprasum*	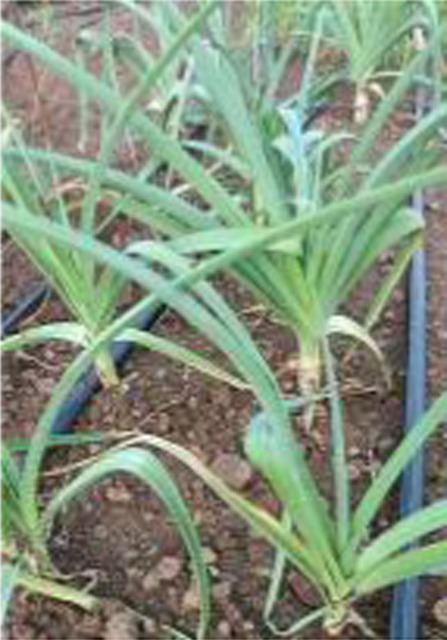 *Allium ampeloprasum*	
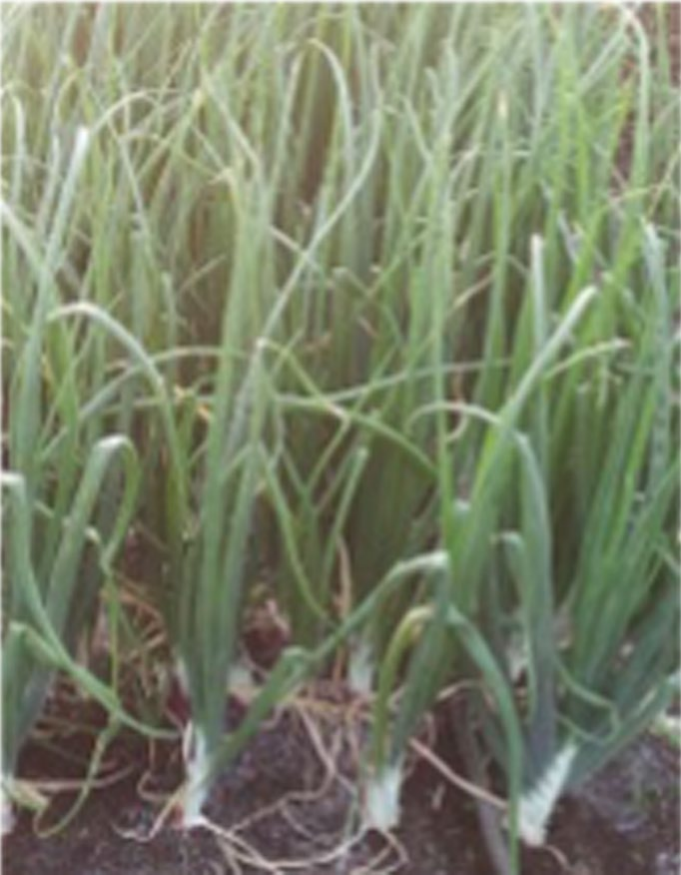 *Allium cepa*	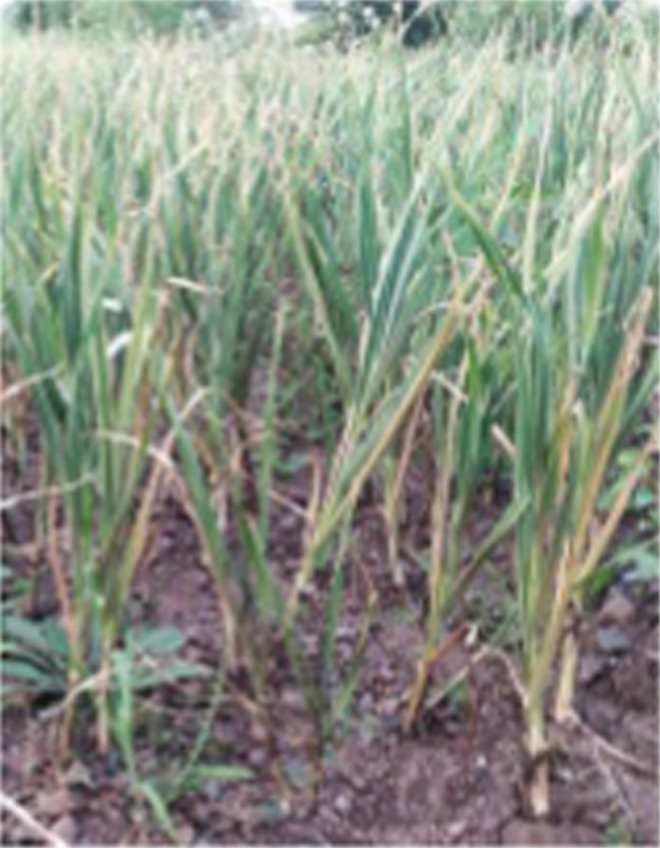 *Allium sativum*	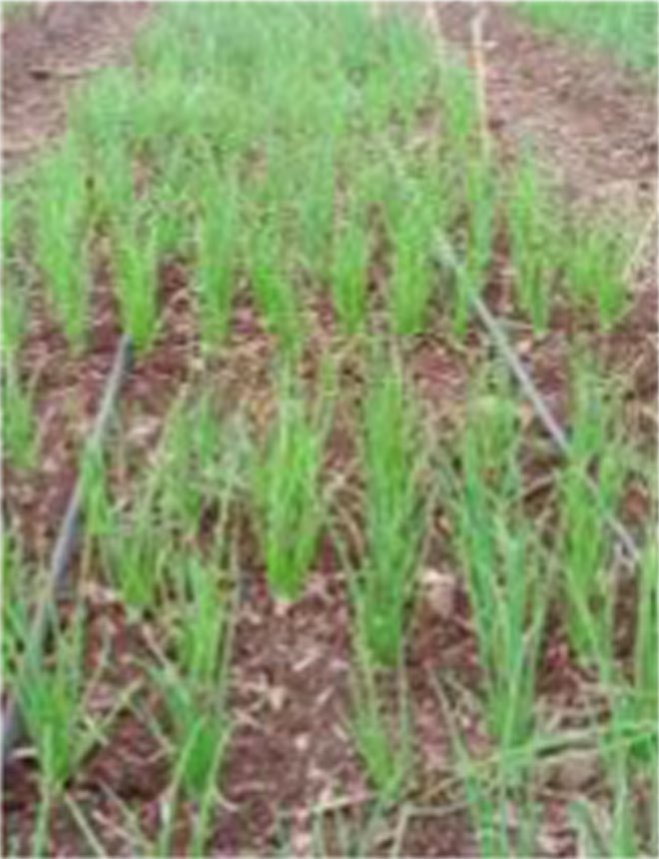 *A. cepa var. aggregatum*		

## Health benefits of *Allium* species

**Figure 1:**
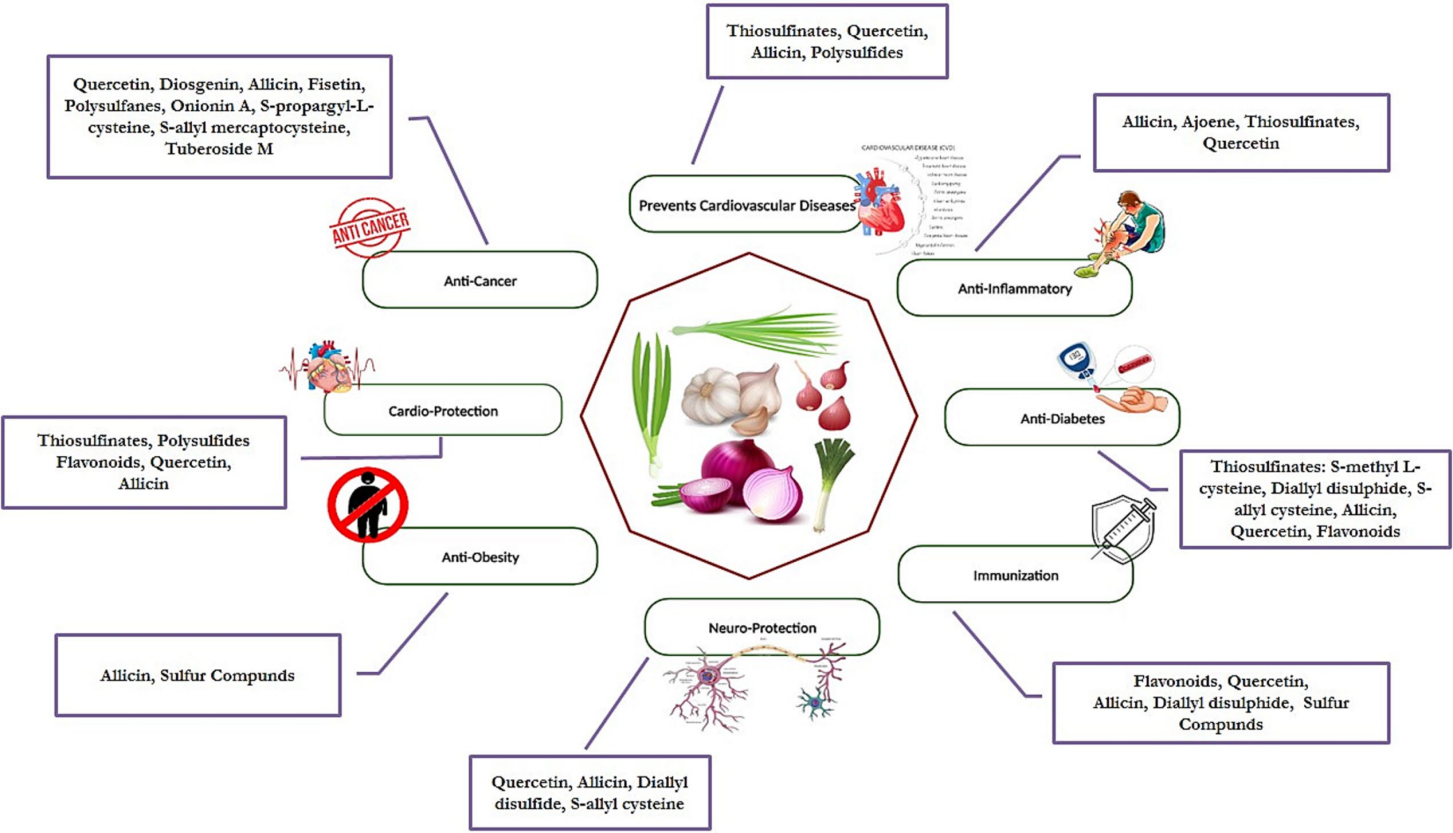
Nutraceutical benefits of Allium species.

Several health benefits of *Allium* spp., including cardiovascular health, enhance immune function, and reduce the risk of cancer has been well documented in various research. Besides, they also help decrease cholesterol, regulate blood pressure, and have antidiabetic qualities. All these are attributed to their antioxidant, anti-inflammatory, and antimicrobial properties. The details of the bioactive and their mode of action is described below (Table 1 and Figure 1).

### Prevention of cardiovascular disease (CVD)

Cardiovascular disease (CVD) is the leading global cause of morbidity and mortality, responsible for 32% of global deaths (17). It is primarily caused by the atherosclerotic plaques or thrombi blocking coronary arteries, as a result of oxidative modification of low-density lipoprotein (LDL) by reactive oxygen species (ROS) contributing to the progression of atherosclerosis (18). Plaque formation constricts the blood vessels the deposition of cholesterol, lipids, and lipoproteins, which can either block arteries or break off and restrict blood flow elsewhere. Besides, increased plasma fibrinogen and platelet aggregation promote thrombus formation, consequently narrowing arteries and causing CVD (19).

Still, CVD can be effectively managed through addressing the major factors such as aberrant cholesterol levels, hypertension, platelet aggregation, and oxidative stress (18). Garlic and onion consumption has been linked to reduce lipid and cholesterol levels (20). Garlic is especially noted for its cardiovascular benefits, such as reducing blood pressure, preventing atherosclerosis, lowering cholesterol and triglycerides, inhibiting platelet aggregation, and increasing fibrinolytic activity (21). Key bioactive compounds in *Allium* spp. such as allicin, flavonoids, and polysulfides in garlic, and quercetin and thiosulfinates in onion contribute to these effects (22, 23). The bioactive compounds in garlic reduces platelet aggregation by inhibiting calcium mobilization (24, 25) and promote fibrinolysis, which aids in dissolution of clots (26). Moreover, these bioactive compounds also scavenges ROS, thereby preventing LDL oxidation (27, 28).

### Anticancerous properties

Globally millions of deaths are reported due to cancer, a complex disease characterized by uncontrolled cell division and the spread of abnormal cells to nearby tissues (29, 30). Asia houses 60% of the global population, and bears nearly half of the global cancer burden (30, 31). Cancer development involves several stages: initiation, promotion, progression, and metastasis (32). The anticancer activity involves multiple cellular mechanisms. Garlic contains allicin, S-allylmercaptocysteine, S-propargyl-L-cysteine, polysulfanes, and other sulfur-based compounds known for their anticancer activity (32–34). Organosulfur compounds, such as allicin, have ability to induce apoptosis (programmed cell death) in cancer cells and inhibit their proliferation. Additionally, the antioxidant properties of the flavonoids help to neutralize harmful free radicals and reduce oxidative stress, a contributing factor in cancer development (35). Several *in vitro* and *in vivo* studies have been documented the anticancerous properties of garlic (29). At early stages of cancer initiation, the bioactive compounds of garlic act as potent antioxidants by scavenging the ROS, promoting detoxification, and aid in DNA repair. This will reduce oxidative stress and suppress the production of tumor-promoting agents like 12-O-tetradecanoylphorbol-13-acetate (TPA). Activating the p53 pathway, allicin could arrest the cell cycle and death in breast cancer cells (36). Furthermore, allicin suppresses the discomfort in oral cancer through inhibiting pain mediators such as endothelin, IL-8, and TNF-*α* (37). Additionally, it can also limit proliferation of cancer cells through suppression of telomerase activity in a dose-dependent manner (38). Likewise, bioactive compounds such as onionin A, fisetin, diosgenin, and quercetin in onion, whereas thiosulfinates and tuberoside M in Chinese chives possess anticancer properties (23). The *in vitro* study on the *A. fistulosum* and *A. sativum* extract, shows the inhibition of the human fibroblasts and keratinocyte growth. This suggests a potential role of these extracts in cancer treatment as they exhibit anti proliferative effects on cancer cells (39).

### Antidiabetic effect

Diabetes mellitus (DM) is a serious global health concern during this modern era, with a projected 325 million cases by 2025 (40). Diabetes is classified into two types: Type 1, an autoimmune disorder in which the pancreas produces little or no insulin, and Type 2, which is characterized by insulin resistance and/or impaired insulin secretion. Predominantly, Type 2 diabetes prevails in 90–95% of the cases reported whereas only 5–10% in Type 1 diabetes. In most cases, it is the children and young adults who are affected than old generation (41). This scenario is due to the sedentary lifestyle, calorie rich foods, obesity, and longer life spans. Hyperglycemia in diabetes is caused by abnormalities in insulin secretion (42), which could be managed effectively through proper diet and enzyme inhibition (43, 44). Alliums especially onion and garlic, have been reported to suppress Type 2 diabetes (45, 46). Functional compounds such as S-methyl L-cysteine, S-allyl cysteine, and diallyl disulphide of garlic while S-methylcysteine and flavonoids of onion possess antidiabetic activities (23). Moreover, polyphenols (allicin) in garlic exhibit both antioxidant and antidiabetic properties, and mimic insulin in glucose metabolism (47). Also, garlic extracts have shown effectiveness in reducing insulin resistance (48). Previous reports suggests that both onion and garlic can inhibit carbohydrate-metabolizing enzymes which results in decreased hyperglycemia in diabetic model animals (49, 50). The butanol fraction of the *A. tuberosum* leaf extract demonstrated significant antidiabetic activity in the alloxan treated mice (14). However the bioactive compounds responsible for this activity remain unidentified, and further research is needed to isolate and characterize these compound. Nevertheless, Quercetin found in onion play a crucial role in inhibiting *α*-glucosidase and enhancing insulin action, along with rutin (51, 52). Besides, sulfur compounds in *Allium* spp. act as free radical scavengers and stimulate insulin secretion (53).

### Osteoporosis (OP)

Osteoporosis (OP) is a hidden, dangerous disease induced by lifestyle choices, resulting in fatal or disabling fractures. One in three women, and one in every 12 males are affected by OP. Among women, 50% over the age of 45, and 90% over the age of 75 are vulnerable. The primary cause include low bone mineral density (BMD) (54), oxidative stress from an imbalance between reactive ROS and antioxidants (55), and an imbalance between osteoclasts and osteoblasts due to estrogen deficiency and inflammation, marked by increased pro-inflammatory cytokines like TNFα and T-cell expression of RANKL (56, 57). Flavonoids, particularly quercetin, and sulfur compounds found in Allium species, have been shown to help reduce osteoporosis. Matheson et al. (58) reported that women who frequently consume onion may lower their risk of hip fractures by over 20% compared to non-consumers. Several preclinical studies have suggested that consumption of onion prevents bone resorption and osteoclast differentiation, hence maintaining normal BMD (59, 60). Quercetin, a phytoestrogen is a potent natural osteogenic agent due to its anti-inflammatory, antioxidant and estrogen-like actions (61–63). It also promotes osteoclast apoptosis and has anti-cancer, anti-depression, and antiviral effects (64, 65). Studies also demonstrate quercetin and its derivatives.

### Antimicrobial, antiviral and anti-inflammatory properties

Antimicrobial and antiviral properties of various *Allium* spp., is attributed to their sulfur rich compounds such as allicin, ajoene, and thiosulfinates. These compounds have exhibited efficacy against a wide range of bacteria, including *Escherichia coli*, *Enterococcus faecalis, Staphylococcus aureus*, *Salmonella typhi, Pseudomonas, Proteus*, and *Helicobacter pylori*, as well as viruses like Influenza (A and B viruses), Rhinovirus and *Herpes simplex virus* (HSV-1 and HSV-2) (66–71). Quercetin in onion is found to be effective against various bacterial and viral infections. Recent studies highlight that allicin disrupts microbial cell walls and inhibits viral replication by targeting essential enzymes. Since the COVID-19 pandemic, there has been interest in the antiviral effects of *Allium* spp. against SARS-CoV-2, the virus responsible for COVID-19 (72). Nevertheless, there is no conclusive evidence that bioactive compounds in garlic inhibit viral proliferation or boost immune response. Therefore, rigorous clinical trials are required to validate these results (73, 74). Similarly, ajoene found in *Allium* spp. possess antifungal properties against various fungi such as *Candida albicans*, *Scopulariopsis* spp., and *Aspergillus* spp. (75). The leaf extract of the *A. tuberosum* highest antimicrobial activity against *Staphylococcus aureus* and *Bacillus subtilis*, compared to *A. sativum* (13). Additionally, the green leaves of *A. fistulosum* contains fructans which possesses potent anti-influenza properties inhibiting the replication of the influenza virus (76). Compounds such as A, B and C isolated from *A. fistulosum* have been found effective against fungal pathogens (77). The *A. schoenoprasum* exhibited antimicrobial activity against strains of *Staphylococcus aureus* and *Bacillus cereus* (78). Moreover, the vitamin C and flavonoids in *A. fistulosum* play a vital role in mitigating inflammatory responses caused by oxidative stress there by reducing inflammation and enhancing body’s defense mechanism (79). The *in vivo* evaluation using a turpentine oil-induced inflammation model in rats has shown that the *Allium schoenoprasum* leaf extract of has inhibitory activity on phagocytosis and oxidative stress (80). Furthermore, ongoing research emphasizes the potential of Allium-derived compounds in combating resistant pathogens (Methicillin-resistant *Staphylococcus aureus* [MRSA] and vancomycin-resistant *Enterococcus* [VRE]) (81).

### Neuroprotective effects

Neurological disorders (Alzheimer’s, Parkinson’s, and brain injuries) are associated with oxidative stress, misfolded proteins, and neuronal loss (82–84). Globally the cases of dementia are expected to hit more 150 million by 2050 (85). Allicin, S-allyl cysteine (SAC), and diallyl disulfide (DADS) in garlic, and quercetin in onion are potent neuroprotective agents. These compounds exhibit antioxidant activities thereby protecting the neurons from oxidative stress, inhibit apoptosis, and reduce inflammation thus preventing neurodegenerative diseases (86). Allicin has the property to enhance cognitive function through restoring neurotransmitter levels, regulating microglial activity and decreasing neuroinflammation (83). Furthermore, *in vitro* studies on mice with cognitive impairments, demonstrated the potential of quercetin in supporting cognitive health by improving behavior and brain redox activity (87).

### Anti-aging effects

Aging, an inevitable biological process often accompanied by reproductive and regenerative decline (88), with skin aging being particularly complex (89, 90). Bioactive compounds present in Allium species are reported to reduce oxidative stress, inflammation, and improve overall cellular health. Allicin in garlic neutralizes free radicals due to its antioxidant effect and protects skin from damaging thereby reduce premature aging (91, 92). Furthermore, it has the potential to inhibit leukocyte elastase thus act as anti-aging agent (93). Likewise, quercetin in onion scavenges free radicals, reduces UV damage, and promotes DNA repair which results in improved skin health (94). Anthocyanins protect the skin from harmful UV radiations while, FOS boost gut health due to their prebiotic activities that eventually leads to healthy aging (95).

## Toxicological implications

Several studies highlight the health benefits of allium and is considered safe for consumption in most individuals. Daily consumption of ½–1 onion and 1–2 garlic cloves (2–5 g) per day is generally considered safe (96). In most individuals, consumption of alliums at large quantities may not affect their metabolism, however in sensitive individuals can exhibit toxic reactions at high doses. Gastrointestinal symptoms such as nausea, vomiting, and diarrhea is often associated with consumption of garlic at large quantities (97, 98). However, it may also cause allergic reactions and skin irritations rarely (99). Long-term consumption of large amounts of garlic may lead to hematologic effects, including bleeding, as garlic inhibits platelet aggregation due to its sulfur compounds. Though garlic is prescribed clinically in the treatment of some disorders, care should be taken regarding its usage with other medications due to possible drug interactions that might arise as a result. Similarly, onion when consumed at large quantities, result in gastrointestinal discomfort and allergic reactions (100). Like any product, consuming large quantities of Alliums can pose risks. It is imperative to consume fresh Alliums in moderation and adhere to recommended supplement dosages to avoid any unintended reactions.

## Challenges and future directions

*Allium* species highlights their potential therapeutic benefits, but several gaps remain that require further investigation. Out of approximately 700 Allium species, only about 30 have been extensively studied. Further research is required to fully explore their bioactive compounds, with particular emphasis on developing new drugs and therapies for carcinogenesis, immune-related, and cardiovascular diseases (101). Extensive work has been done on substantiating the health benefit of bioactive compounds such as allicin and quercetin, whereas several other phytochemicals remain underexplored. Most studies focused on their individual effects in preventing and alleviating these lifestyle diseases, however their synergistic action in complex mixtures are not fully understood. Furthermore, low stability and bioavailability of these compounds poses challenges for deciding the recommended dose. Similarly, the precise molecular mechanisms underlying their mode of action remain unclear. It is imperative to gain a deeper understanding of how these compounds are absorbed, metabolized, and excreted in the human body, as well as their precise effects on cellular pathways and gene expression.

Besides the uncertainty in dosage and physiological pathways, extraction of specific bioactive compound and its preservation using modern extraction methods such as steam distillation and solvent extraction poses significant challenge. Technologies like supercritical fluid extraction (SFE), ultrasound-assisted extraction (UAE), and microwave-assisted extraction (MAE), improved the yield and purity of extracted bioactive compound. With the advent of advanced chromatographic techniques like High-performance liquid chromatography (HPLC) and molecular imprinting, the selective isolation of bioactive compounds has become increasingly feasible. These techniques help to identifying and isolating the precise compound from the mixture, contributing to the development of effective drugs for therapeutic interventions. Most bioactive compounds are sensitive to heat, light, and oxygen, leading to degradation during extraction and storage. For instance, allicin is highly unstable and degrades rapidly upon oral consumption due to its interaction with stomach acids (102). Therefore, innovative stabilization methods such as encapsulation is required to safeguard these heat- and oxygen-sensitive bioactive compounds during extraction and storage. Currently, traditional polymers and lipids are used for encapsulation. However, future research should focus on developing novel materials that both are biocompatible and biodegradable, aligning with sustainability goals. Furthermore, enhancing the loading capacity of encapsulation systems and developing target specific controlled-release formulations could maximize therapeutic efficacy while improving bioavailability. Scaling up encapsulation techniques for industrial applications is crucial. Advanced methods such as nanocarriers, coacervation, and electrospinning hold significant promise for achieving large-scale production while preserving the stability and efficacy of bioactive compounds. These approaches offer enhanced efficiency and scalability, ensuring that the encapsulated materials retain their desired properties during processing and storage.

Robust human clinical trials to compare the bioavailability and efficacy of encapsulated versus non-encapsulated forms is crucial for validating their therapeutic potential. Furthermore, while research on *Allium* spp. has demonstrated their health benefits, particularly in cancer and chronic disease management, more studies are needed to investigate their long-term effects and safety, especially in relation to genetic variability and interactions with other medications. Genetic differences and dietary habits among individuals can influence the response to Allium-derived compounds, underscoring the need for personalized health strategies. To fully harness the therapeutic potential of *Allium* spp., it is essential to develop sustainable cultivation practices that ensure a consistent supply of high-quality products. Integrating *Allium* spp. into public health recommendations and dietary guidelines could also promote their use for disease prevention and health promotion.

The increasing interest in Allium supplementation to enhance health benefits requires several factors to be considered like supplementation dose, formulation and delivery, clinical trials, personalized approaches and dietary integration. For instance, in most clinical studies, the daily dose of dehydrated garlic powder has been approximately 900 mg. 1–7.2 g/d of Aged garlic extract (AGE) which contains allicin is often recommended for various health benefits including cardio vascular health. Studies also showing that 1.8–10 g/d of AGE have immune enhancement in humans (103). However, development of bioavailable formulation of Allium supplementation is crucial as Allicin is highly unstable and degrade quickly due to which garlic supplementation often undergoes like aging to preserve its active ingredients. Furthermore, clinical trials to evaluate the side effects of Allium supplements administered for various health conditions is important.

Harnessing the nutraceutical and therapeutic potential of *Allium* spp. also supports several Sustainable Development Goals (SDGs). By offering plant-based treatments for non-communicable diseases like cardiovascular conditions and cancer, they contribute to SDG 3: Good Health and Well-being. As nutrient-rich crops, Allium species can be integrated into food security programs, aligning with SDG 2: Zero Hunger. Their cultivation promotes eco-friendly practices, reducing reliance on synthetic drugs and pesticides, contributing to SDG 12: Responsible Consumption and Production. Additionally, Allium plants require low environmental input, supporting sustainable agriculture and resilience to climate change, which ties into SDG 13: Climate Action. In summary, advancing research on *Allium* spp. requires a multifaceted approach, combining novel extraction and encapsulation methods, detailed molecular studies, and large-scale clinical trials to fully understand and optimize their therapeutic potential.

## Data Availability

The original contributions presented in the study are included in the article/supplementary material, further inquiries can be directed to the corresponding authors.
